# Knockout of HDAC9 Gene Enhances Foot-and-Mouth Disease Virus Replication

**DOI:** 10.3389/fmicb.2022.805606

**Published:** 2022-02-18

**Authors:** Shitong Hou, Xiangwei Wang, Shanhui Ren, Xuelian Meng, Xiangping Yin, Jie Zhang, Kazimierz Tarasiuk, Zygmunt Pejsak, Tao Jiang, Ruoqing Mao, Yongguang Zhang, Yuefeng Sun

**Affiliations:** ^1^State Key Laboratory of Veterinary Etiological Biology, National Foot and Mouth Diseases Reference Laboratory, Key Laboratory of Animal Virology of Ministry of Agriculture, Lanzhou Veterinary Research Institute, Chinese Academy of Agricultural Sciences, Lanzhou, China; ^2^Hebei Normal University of Science and Technology, Qinhuangdao, China; ^3^University Center of Veterinary Medicine JU-AU, Krakow, Poland

**Keywords:** HDAC9, FMDV, CRISPR/Cas9, RNA-Seq, innate immune response

## Abstract

Foot-and-mouth disease virus (FMDV) is a highly contagious viral disease that mainly infects cloven-hoofed animals. Propagation of FMDV by cell culture is an important method to preserve viral biological and antigenic characteristics, which is crucial in FMD monitoring and vaccine production. However, only a few cell lines are sensitive to FMDV, and there is still a lot of room for improvement. Acetylation is an important post-translational modification, which is dynamically regulated by histone acetyltransferases (HATs) and histone deacetylases (HDACs). However, the study of the relationship between FMDV and HDACs is still unclear. HDAC9 belongs to the class II of HDACs family; in this study, HDAC9 knockout (KO) BHK-21 cells were successfully established using CRISPR/cas9 technology. The results of karyotype analysis, growth curve analysis, and morphological observation showed that the HDAC9 knockout cell line was stable in growth and morphological characteristics. After infection with FMDV, the expression of viral RNA and protein, viral titers, and the copies of viral RNA in HDAC9-KO cells were significantly higher than those in NC cells. Meanwhile, RNA-seq technology was used to sequence HDAC9-KO cells and NC cells infected and uninfected with FMDV. It was found that the differentially expressed innate immune factors containing NFKBIA, SOD2, IL2RG, BCL2L1, CXCL1/2/3, and IL1RAP have significantly enriched in the Jak-STAT, NOD-like receptor, Toll-like receptor, NF-κB, and MAPK signaling pathway. RT-qPCR was performed to detect the expression level of differentially expressed genes and showed consistency with the RNA-seq data. These results preliminarily reveal the role of HDAC9 in host antiviral innate immune response, and the HDAC9-KO cell line could also serve as a useful tool for FMDV research.

## Introduction

Foot-and-mouth disease (FMD) is an acute, highly contagious infectious disease caused by foot-and-mouth disease virus (FMDV), which mainly infects cloven-hoofed livestock and wild animals (Alexandersen and Mowat, 2005). The disease is characterized by vesicular lesions in the buccal cavity, feet, and teats (Grubman and Baxt, 2004), causing severe damages to agricultural development. FMDV belongs to the member of the *Aphthovirus* genus within the family *Picornaviridae*. Its genome is a positive single-strand RNA virus with a total length of about 8.4 kb. FMDV has seven different serotypes, named O, A, C, Asia 1, SAT1, SAT2, and SAT3. In addition, multiple subtypes were further evolved from each serotype (Knowles and Samuel, 2003). FMD vaccine development faces many challenges because there is no cross-protection between different serotypes (Klein, 2009).

Acetylation is an important post-translational modification, including histone and non-histone acetylation (Glozak et al., 2005). Previous studies have shown that protein acetylation affects the infection and immune process of virus in many ways, such as viral protein acetylation, histone acetylation in the promoter region of host immune-related genes, and acetylation of immune signaling molecules (Kiernan et al., 2003; Nusinzon and Horvath, 2003; Choi et al., 2016; Hatakeyama et al., 2018). The levels of protein acetylation are dynamically regulated by histone acetyltransferase (HAT) and histone deacetylase (HDAC). There are 18 members of the mammalian HDACs family. At present, 18 different HDACs are divided into four categories: Class I is HDAC1, 2, 3, and 8, mainly located in the nucleus; Class II is HDAC4, 5, 6, 7, 9, and 10, which can shuttle between nucleus and cytoplasm; Class III is members of SIRT family, including Sirt 1–7; Class IV is HDAC 11, mainly located in the nucleus (de Ruijter et al., 2003). Different HDACs have specific functions without obvious functional redundancy. Individual members of the HDACs family have been shown to play important regulatory roles in the process of virus–host interactions (Feng et al., 2018). Previous studies have shown that methyltransferase Dnmt3a upregulates HDAC9 to deacetylate the kinase TBK1 for activation of antiviral innate immunity (Li et al., 2016). However, the role of protein acetylation and HDACs family genes in FMD infection is unclear.

BHK-21 (baby hamster kidney) cell lines and PK-15 (porcine kidney) cell lines (Zhang et al., 2018) have been successfully used for FMDV research and vaccine development. BHK-21 cells grow rapidly and have a wide sensitive spectrum of viruses. Subsequently, it was used for the proliferation of various viruses and vaccine production, such as FMD vaccine, rabies vaccine, and Newcastle disease vaccine (Amadori et al., 1997).

In this study, HDAC9-KO cell lines were established by using CRISPR/cas9 technology and identified by a series of experiments. Furthermore, the RNA-seq technology was used to reveal the signal transduction pathway of host–virus interaction at the mRNA level during FMDV infection. The differentially expressed genes (DEGs) were analyzed between FMDV-infected and non-infected NC and HDAC9-KO cells. Kyoto Encyclopedia of Genes and Genomes (KEGG) pathways were significantly enriched in the different signaling pathways; the typical genes associated with immune pathways were selected for verification by real-time quantitative PCR (RT-qPCR).

## Materials and Methods

### Cell and Viruses

Baby hamster kidney cells (BHK-21 cell) and porcine kidney cells (PK-15 cell) were maintained in Dulbecco’s modified Eagle’s medium (DMEM) supplemented with 10% fetal bovine serum (FBS) and 1% penicillin–streptomycin in a humidified incubator at 37°C with 5% CO_2_.

FMDV O/BY/2010 strain is stored in the National FMD Reference Laboratory (Lanzhou, Gansu, P.R. China). FMDV was propagated in BHK-21 cells, and the virus titers were determined by 50% tissue culture infectious dose (TCID_50_), which was calculated by the Reed-Muench formula. All virus-related experiments were conducted in the Biosafety Level-3 (BSL-3) Laboratory of Lanzhou Veterinary Research Institute following the standard protocols and biosafety regulations provided by the Institutional Biosafety Committee.

### Establishment of HDAC9 Knockout BHK-21 Cell Line

HDAC9-Knockout (KO) cell lines were established by using the CRISPR/cas9 system following the published protocols (Ran et al., 2013; Haeussler et al., 2016). To establish the HDAC9-KO cell lines, two pairs of the small guide RNAs (sgRNA) targeting the HDAC9 gene of BHK-21 cells were designed using the CRISPOR tool.^1^ Two sgRNAs were annealed and ligated to pspcas9 (BB) plasmid. The molecularly confirmed plasmids were transfected into BHK-21 cells, 0.3 μg puromycin was added per 1 mL of DMEM supplements, and the medium was renewed every 2 days. Single-cell clone was selected by clone ring anchoring method after puromycin selection. The obtained monoclonal cell lines were identified by sequencing and Western blotting. The primers used for plasmid construction and sequencing are listed in Table 1.

**TABLE 1:** The primers of HDAC9 used for plasmid construction and sequencing.

Gene	Sequence (5′→3′)
HDAC9-g26-fw	CACCGGTGAAGTCCGAGGTTCCGAT
HDAC9-g26-rv	AAACATCGGAACCTCGGACTTCACC
HDAC9-g26GT-FP	ACGTTGGTTAAGTGGTCCTGT
HDAC9-g26GT-RP	GGTCAGGTTCTCATGCTGCT
	

### Western Blotting

Cell precipitate was lysed in RIPA Lysis Buffer containing a 1% protease inhibitor cocktail. Total protein concentrations were quantified using the BCA Protein Assay kit. Equal amounts of protein samples were separated by sodium dodecyl sulfate-polyacrylamide gel electrophoresis (SDS-PAGE) and transferred to polyvinylidene difluoride (PVDF) membrane. Then, the membrane was blocked with 5% skim milk at room temperature for 1 h, and the membranes were incubated with primary antibody overnight at 4°C. After that, the membrane was washed with TBST three times before incubation with HRP-conjugated secondary antibody for 1 h followed by chemiluminescent detection.

### Virus Infection, RNA Extraction, and RT-qPCR

BHK-21 cells were cultured in a 60-mm dish to reach an approximate 90% confluence, which was washed once with PBS and incubated with FMDV at a multiplicity of infection (MOI) of 0.1 at 37°C for 1 h. Then, the cells were washed again with PBS and cultured in 3 mL of FBS-free DMEM. After infection, the supernatant was removed and 1 mL of TRIzol Reagent (Invitrogen) was added to each dish. Total RNA was isolated according to the manufacturer’s instructions. RNA (1 μg) was used as the template for cDNA synthesis using PrimeScript™ RT reagent Kit with gDNA Eraser (TAKARA). cDNA was then subjected to real-time PCR quantification using SYBR green Premix Ex Taq II (TAKARA). β-Actin gene was used as an internal control. The primers used in the experiment are listed in Table 2. All the experiments were repeated at least three times, and relative mRNA expression levels were calculated using the threshold cycle (2^–△^^△^ *^Ct^*) method.

**TABLE 2:** Primers used for RT-qPCR amplification.

Gene	Sequence (5’→3’)
VP1-qFP	GACAACACCACCAACCCA
VP1-qRP	CCTTCTGAGCCAGCACTT
3D-qFP	ACTGGGTTTTACAAACCTGTGA
3D-qRP	GCGAGCCCTGCCACGGA
β-Actin-qFP	GCTGGCCGGGACCTGACAGACTACC
β-Actin-qRP	TCTCCAGGGAGGAAGAGGATGCGGC
NFKBIA-qFP	ACCAACTACAACGGCCACA
NFKBIA-qRP	GGCCCCAGGTAAGCTGATAG
BCL2L1-qFP	AATCAGCTCGCAGATCCGAA
BCL2L1-qRP	GAGATGGGCTCAACCAGTCC
CYLD-qFP	AGGCCTATGGAGCCAAGAGA
CYLD-qRP	GCCTTTTGCGGAAGGAACTC
CALM1-qFP	ACGATTGAGCACAGTCAGGC
CALM1-qRP	TTCTGGGGCTGTGTCTCCAA
IGFBP5-qFP	GTTTGTTTGGGTGAGGGCAC
IGFBP5-qRP	CTTTCTCATCGCAGGGCTCA
CXCL1/2/3-qFP	CCAGCACCTCAACTCCAGAC
CXCL1/2/3-qRP	GGTGAACCCCTGTCATGGTC
CCL20-qFP	TCCGTGTGTGCTGATCCAAA
CCL20-qRP	GTCTGTGCAATGACGTGCAG
SOD2-qFP	GAGCCCTAATGGTGGTGGAG
SOD2-qRP	GCAGCAATCTGTAAGCGACC
PLEKHA4-qFP	ACCACATCGTCTGTGAGCAG
PLEKHA4-qRP	CTGACACTGTAGCTGGGCAA
IL2RG-qFP	AATCTTGTGATCCCCTGGGC
IL2RG-qRP	ACAGAAGGATTCTAGTTTCTGTCCA
BIRC3-qFP	ATTTAAAGGCGTCGTGGCG
BIRC3-qRP	TACTAGGCTGAACACCGCAG
TRAF2-qFP	TCGAGGGTGCTGCTCTAGTA
TRAF2-qRP	GGGAAAGCAAGCCACCAAAG

### Growth Characteristics of Foot-and-Mouth Disease Virus in HDAC9 Knockout Cells

The distribution of FMDV in HDAC9-KO cells was detected by indirect immunofluorescence assay (IFA). NC and HDAC9-KO cells were infected with FMDV at an MOI of 0.1 for 6 h. The cells were washed 3 times and fixed with 4% paraformaldehyde for 1 h, permeabilized with 0.1% Triton X-100 for 5 min, and blocked with 3% BSA for 1 h. The cells were incubated with anti-VP1 antibody (1:500 dilution by 1% BSA) at 4°C overnight. Then, the cells were incubated with goat anti-rabbit IgG-FITC antibody (1:500 dilution by1% BSA) at 37°C for 1 h, and the nuclei were stained with DAPI. Finally, the cells were observed by confocal microscopy.

The growth characteristics of FMDV in NC and HDAC9-KO cells were detected by qRT-PCR. HDAC9-KO cells and NC cells were seeded in 24-well plates (5 × 10^5^ cells/well) for 24 h and then infected with the FMDV at an MOI of 0.001 when confluence reached 90%. Viral genome RNAs were extracted using a Viral RNA kit (Omega). The platinum Quantitative RT-PCR kit was used for the qRT-PCR assay, and the primer and probe sequence are shown as the supporting reference (Mao et al., 2018). All the experiments were repeated at least three times.

### TCID_50_ Assay

Virus titers were determined using TCID_50_ assay. BHK-21 cells were seeded in 96-well plates with 90% confluence, and a series of 10-fold serial dilutions from 10^–1^ to 10^–8^ of virus samples were prepared in another plate. One hundred microliters of the above samples were added to each well, and the plates were incubated at 37°C for 1 h. Then, the inoculum was removed and cells were cultured in DMEM supplemented with 1% FBS for 72 h. All plates were analyzed by microscopic examination to determine the cytopathogenic effect (CPE). TCID_50_ was calculated by the Reed–Muench method (Reed and Muench, 1937).

### Transcriptome RNA-Seq

The differential expression of mRNA in FMDV-infected and uninfected HDAC9-KO cells and NC cells was detected to identify the pathways of innate immunity using RNA sequencing (RNA-seq). The cells were infected with FMDV at an MOI of 0.1 for 6 h. The samples were washed three times with PBS, and total RNA was extracted using TRIzol Reagent. These samples were analyzed at the transcriptome level using RNA-seq at BGI Genomics (Wuhan, China). The data were obtained by sequencing named raw reads and then quality control (QC) was performed. High-quality reads were aligned to the reference genome. After that, gene quantitative analysis and various analyses based on gene expression level (principal component, correlation, differential gene screening, etc.) were carried out, and the DEGs between samples were analyzed. Fragments per kilobases per million fragments method (FPKM) was used to evaluate the expression level of Unigene and the fold change between different samples. The DEGs were selected with log_2_ (fold change) > 1 or log_2_ (fold change) < −1 and statistical significance (*p*-value < 0.05).

### Karyotype Analysis

HDAC9-KO cells and NC cells were treated with 0.1 μg/ml colchicine at 37°C for 6 h to obtain metaphase cells, which were harvested and the numbers of chromosomes were determined. Then, the G-banding technique is used to identify individual pairs of chromosomes based on size and space.

### Cell Proliferation Assay

Cell proliferation was assayed using Cell-Counting Kit 8 (CCK8) at the indicated time points. HDAC9-KO cells and NC cells were seeded in 96-well plates (1 × 10^4^ cells/well). To measure the cell viability at the indicated time points, CCK8 reagent was added to each well for 10 μL and incubated for 1 h. After that, the absorbance at 450 nm was determined with Varioskan™ LUX multimode microplate reader (Thermo).

### Statistical Analysis

All the experiments were repeated at least three times, the statistical significance was evaluated by Student’s *t*-test using the SPSS 17.0 software.

## Results

### Successfully Established HDAC9 Knockout BHK-21 Cell Lines

**FIGURE 1:**
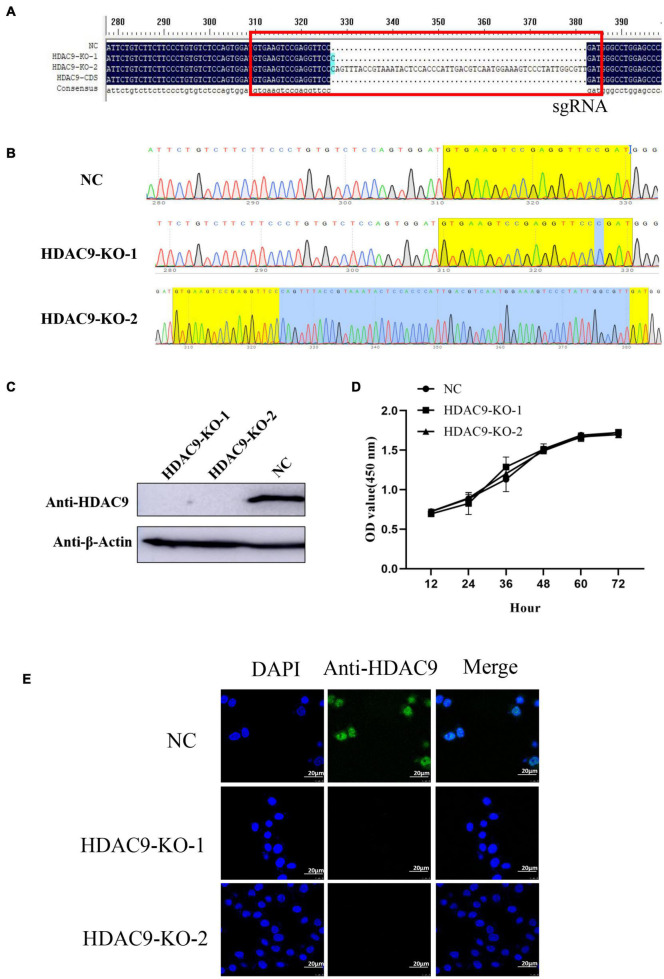
Identification of the HDAC9 knockout BHK-21 cells. **(A)** Alignment of the NC (cells transfected with empty vector and selected by puromycin), HDAC9-KO-1, HDAC9-KO-2, and HDAC9 CDS sequences using DNAMAN software. The red box indicates the sgRNA sequence and the mutations in PAM motif. **(B)** Snapgene software was used to confirm the sequencing peaks of PCR products of NC, HDAC9-KO-1, and HDAC9-KO-2. **(C)** The expression of HDAC9 was detected by Western blotting with HDAC9 antibody, and β-Actin was used as a control to show the even loading of samples. **(D)** NC and HDAC9-KO cells (HDAC9-KO-1 and HDAC9-KO-2) were seeded in 96-well plates at 1 × 10^4^ cells/well. CCK8 reagent was added to each well for 10 μL and incubated for 1 h, and then the absorption at 450 nm was determined. **(E)** Immunofluorescence assay of NC and HDAC9-KO cells (HDAC9-KO-1 and HDAC9-KO-2); the signals of HDAC9 protein were observed under a confocal microscope. Green signals represent HDAC9 and nucleus was stained with DAPI. Statistical significance was analyzed by Student’s *t*-test: *p < 0.05, **p < 0.01.

HDAC9 knockout (HDAC9-KO) BHK-21 cells were established using the CRISPR/cas9 system. Two pairs of the small guide RNAs (sgRNA) were designed by the CRIPOR tool and ligated to the pspcas9 (BB) plasmid. Single-cell clones were picked using the cloning-ring anchoring method and identified after extended culture. The gene of HDAC9 in the cell line was confirmed by DNA sequencing. The results showed that one nucleotide insertion was detected in the first exon of one allele of HDAC9 (HDAC9-KO-1), and a 55-nucleotide insertion was introduced into the other allele of HDAC9 (HDAC9-KO-2) (Figures 1A,B). The expression of HDAC9 in the cell line was confirmed by Western blotting. The results showed that HDAC9 was successfully knocked out in two BHK-21 cell lines compared with the NC cell line (Figure 1C). Then, the cell growth curve was created and the results showed that there was no significant difference in growth rate between HDAC9-KO cell lines (HDAC9-KO-1 and HADC9-KO-2) and NC [cells transfected with empty pspcas9 (BB) plasmid and selected by puromycin] (Figure 1D), suggesting that the HDAC9-KO cell lines could be extended indefinitely with steady growth rate. As shown in Figure 1E, HDAC9 was mainly distributed in the nucleus, and more green fluorescence signals were observed in NC cells compared with HDAC9-KO cells.

**FIGURE 2:**
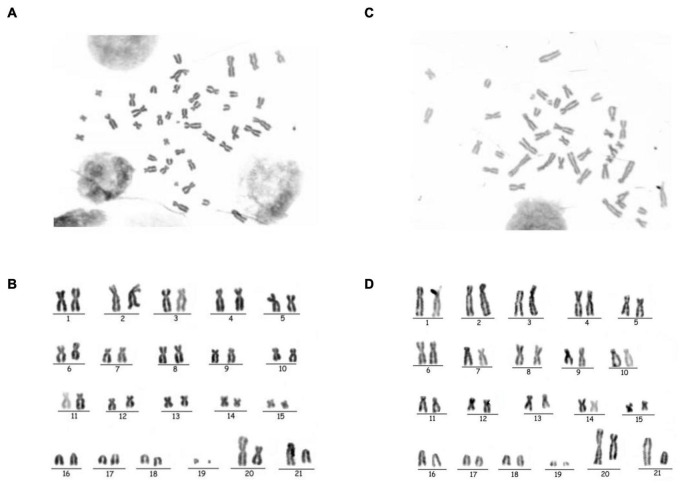
Chromosomal analysis of NC and the HDAC9-KO-1 cell lines. **(A)** Chromosomes of NC with a diploid number of 42. **(B)** GTG-banded NC Karyotype. **(C)** Chromosomes of HDAC9-KO-1 with a diploid number of 42. **(D)** GTG-banded HDAC9-KO-1 karyotype.

Both structural and number variations in chromosomes were detected using karyotype tests. Chromosome numbers of HDAC9-KO-1 cells and wild-type BHK-21 cells were analyzed. We found no evidence for an absolute difference between chromosome numbers in HDAC9-KO-1 cells and wild-type BHK-21 cells, which both have 42 chromosomes (Figures 2A,C). The G-banding technique was used to confirm whether the process of gene knockout induced chromosomal structural aberrations of HDAC9-KO-1 cells. As shown in Figures 2B,D, all chromosomes were unchanged in the HDAC9-KO-1 cells, and the cell line had the same karyotype as the wild-type BHK-21 cells without chromosomal aberration.

### HDAC9 Knockout Enhances Foot-and-Mouth Disease Virus Replication

**FIGURE 3:**
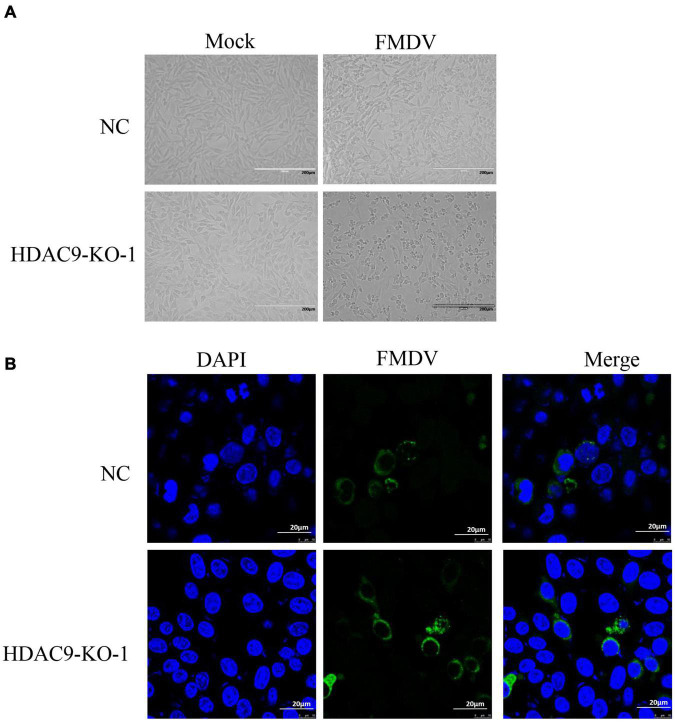
Growth characteristics of FMDV. **(A)** CPE caused by FMDV, NC, and HDAC9-KO-1 cells was seeded in 6-well plates (2 × 10^6^ cells/well) and were infected with FMDV at an MOI of 0.1 for 4 h. **(B)** Immunofluorescence assay of NC and HDAC9-KO-1 cells infected with FMDV. The cells were infected with or without FMDV at an MOI of 0.1. After 6 h, the signals of FMDV protein were observed under a confocal microscope. Green signals represent FMDV and nucleus was stained with DAPI.

NC and HDAC9-KO-1 cells were infected with FMDV at an MOI of 0.1. The cytopathic effect (CPE) could be observed at 4 h after virus infection, and more CPE was observed in HDAC9-KO-1 cells (Figure 3A). Indirect immunofluorescence experiments were carried out to further visualize the distribution of FMDV in NC and HDAC9-KO-1 cells. As shown in Figure 3B, viral structure protein VP1 was mainly distributed in the cytoplasm and more green fluorescence signals were observed in HDAC9-KO-1 cells compared with NC cells.

**FIGURE 4:**
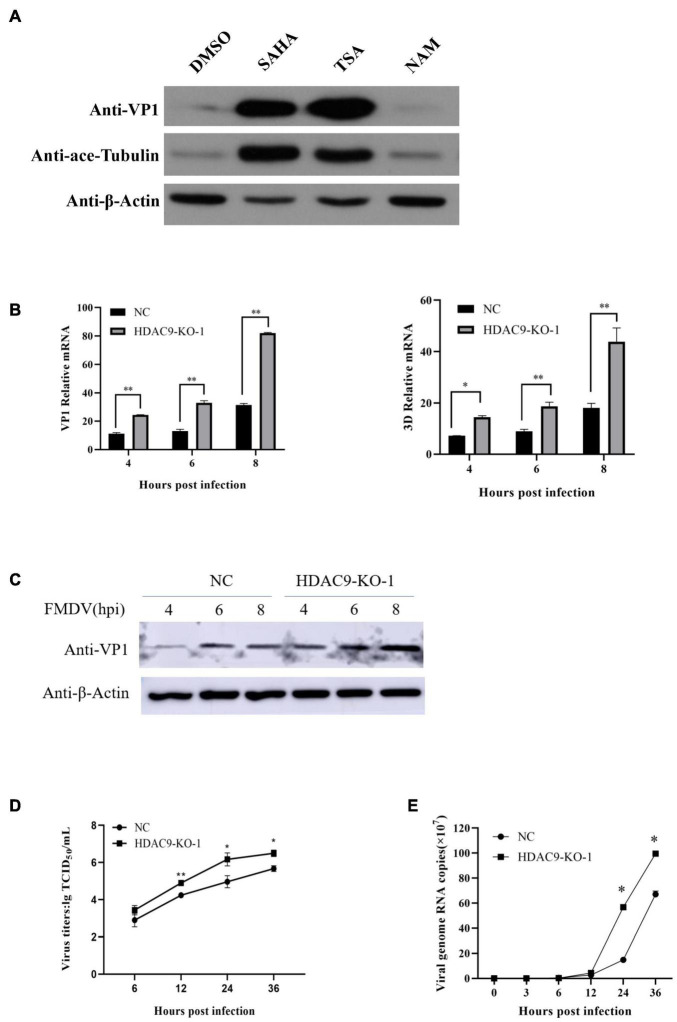
HDAC9 knockout enhances FMDV replication. **(A)** BHK-21 cells were seeded in 60-mm dishes to reach an approximate 90% confluence and infected with FMDV at an MOI of 0.1. Cells were incubated for 1 h and treated with HDAC inhibitors (TSA, SAHA, and NAM), respectively. Samples were collected at 6 hpi. VP1 protein and ace-tubulin were determined by Western blot with indicated antibodies. **(B)** HDAC9 knockout cells (HDAC9-KO-1) and NC were infected with equal amounts of FMDV at an MOI of 0.1 for 4, 6, and 8 h. qRT-PCR was performed to examine the expression of FMDV VP1 and 3D relative mRNA. **(C)** The cells were infected with equal amounts of FMDV at the same time as in **(B)**. The expression of FMDV VP1 protein was determined by Western blot, and β-Actin was used as an internal reference. **(D)** FMDV titers were determined by the TCID_50_ method after cells were infected with FMDV at an MOI of 0.001. **(E)** NC and HDAC9-KO cells were separately seeded in 24-well plates (5 × 10^5^ cells/well), and the cells were infected with virus at an MOI of 0.001. Cells and supernatants were collected 0, 3, 6, 12, 24, and 36 hpi, and the expression of viral RNA was determined using qRT-PCR. All experiments were repeated three times, with similar results. Statistical significance was analyzed by Student’s *t*-test: *p < 0.05, **p < 0.01.

TSA (trichostatin A) and SAHA (vorinostat) are broad-spectrum HDAC inhibitors, and NAM is an inhibitor of SIRT family. As shown in Figure 4A, the expression of VP1 protein increased significantly after TSA and SAHA treatment. This suggests that HDACs rather than SIRT families can regulate FMDV replication. Equal amounts of FMDV (MOI = 0.1) were incubated with NC and HDAC9-KO-1 cells; the viral mRNA levels of structural protein VP1 and non-structural protein 3D, and the expression of VP1 protein and viral titers were determined and compared. As shown in Figures 4B–D, the expression of viral RNA and protein, as well as the viral titers, was significantly higher in HDAC9-KO-1 cells compared to the NC cells. Then, total viral RNA of FMDV (MOI = 0.001) was collected, and the viral growth curve was detected by qRT-PCR. As shown in Figure 4E, the number of RNA copies of the FMDV increased acutely from 6 h post-infection, and the growth rate is the fastest in 12–36 h. It was also found that compared with NC cells, the HDAC9-KO-1 cells had a higher number of viral copies and a faster viral replication rate. These results showed that knockout of HDAC9 gene enhances FMDV replication in the BHK-21 cell line.

**FIGURE 5:**
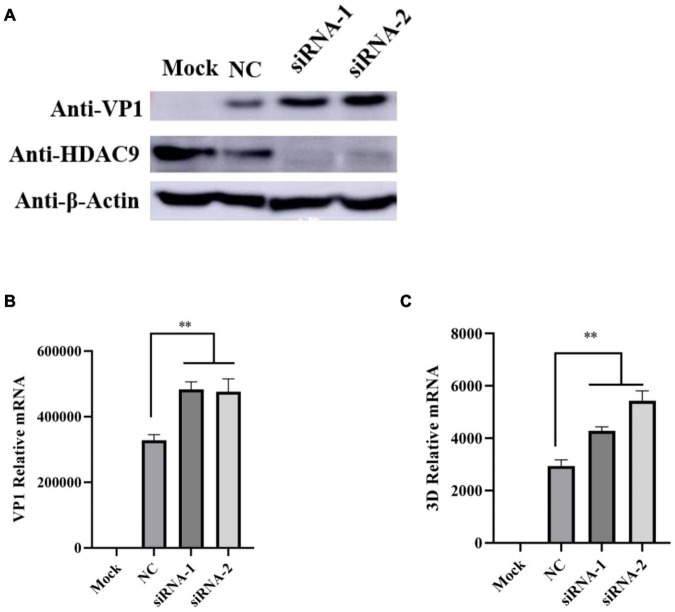
Knockdown of HDAC9 enhances FMDV replication in PK-15 cells. PK-15 cells were transfected with control siRNA (NC) or siRNA targeting to HDAC9 for 24 h, and then infected with FMDV (MOI = 1) for 6 h. **(A)** Western blot analysis was performed to detect the expression of HDAC9 and viral proteins VP1 with indicated antibodies. **(B,C)** Total RNA was extracted and qRT-PCR was performed to detect the expression of FMDV VP1 and 3D relative mRNA. All experiments were repeated three times, with similar results. Data are presented as mean ± SD (*n* = 3). Statistical significance was analyzed by Student’s *t*-test: *p < 0.05, **p < 0.01.

To further confirm the above results, RNA interference technique was performed in PK-15 cells. The knockdown effects were confirmed by Western blot analysis (Figure 5A). As shown in Figures 5A–C, compared with the control siRNA transfected cells (NC), the viral protein level, VP1 and 3D mRNA levels were significantly increased. These results indicated that the knockdown of HDAC9 by siRNA significantly enhanced FMDV replication in PK-15 cells.

### Foot-and-Mouth Disease Virus Triggered a Lesser Degree Anti-Virus Innate Immune Response in HDAC9-KO Cells

RNA-seq technology was used to sequence the gene expression of NC and HDAC9-KO-1 cells infected with FMDV or not. The relevant clean data have been uploaded to the NCBI database.^2^ The sequencing generated a total of 261 million raw sequencing reads and 259.75 million clean reads (99.52% of total reads). To investigate the DEGs in NC and HDAC9-KO-1 cells after FMDV infection, an independent statistical hypothesis test was used to detect all DEGs, and the different test *p*-value was provided. Meanwhile, the DEGs were used to perform GO enrichment analysis.

**FIGURE 6:**
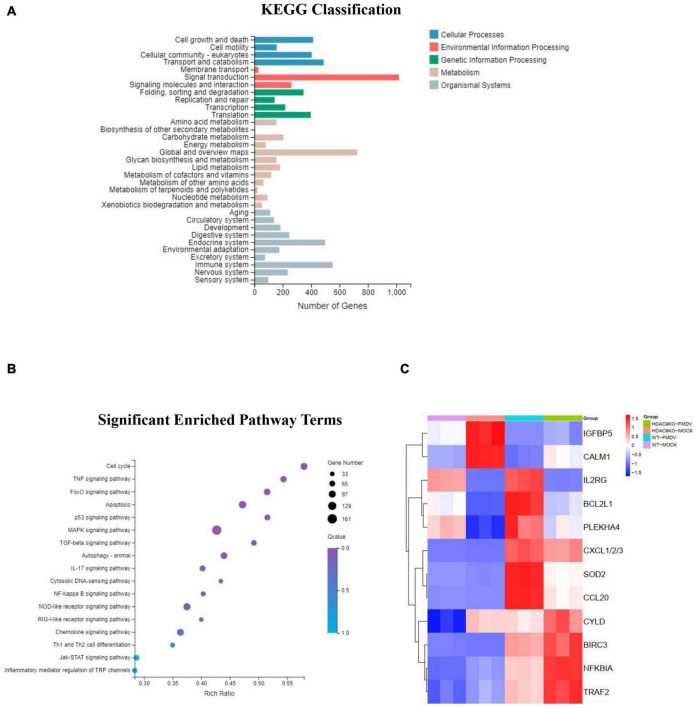
KEGG classification and pathway enrichment analysis of DEGs. **(A)** KEGG classifications of DEGs. The *x*-axis indicates the number of DEGs and the *y*-axis indicates the different terms. **(B)** Significant enriched pathway results for the DEGs. The *x*-axis indicates the rich factor of GO and the *y*-axis indicates the GO term (the recommended cutoff of *p*-value is 0.05). **(C)** Heatmap of selected differentially expressed genes involved in immune response.

As shown in Figures 6A,B, KEGG classification showed that the DEGs were mainly involved in signal transduction, immune system, endocrine system, transport and catabolism, cell growth, and death. The KEGG pathways were significantly enriched in the TNF, FoxO, p53, MAPK, IL-17, NF-κB, NOD-like receptor, Toll-like receptor, and RIG-I-like receptor signaling pathway. These pathways were mainly regulated to FMDV infection triggered anti-virus innate immune response.

**FIGURE 7:**
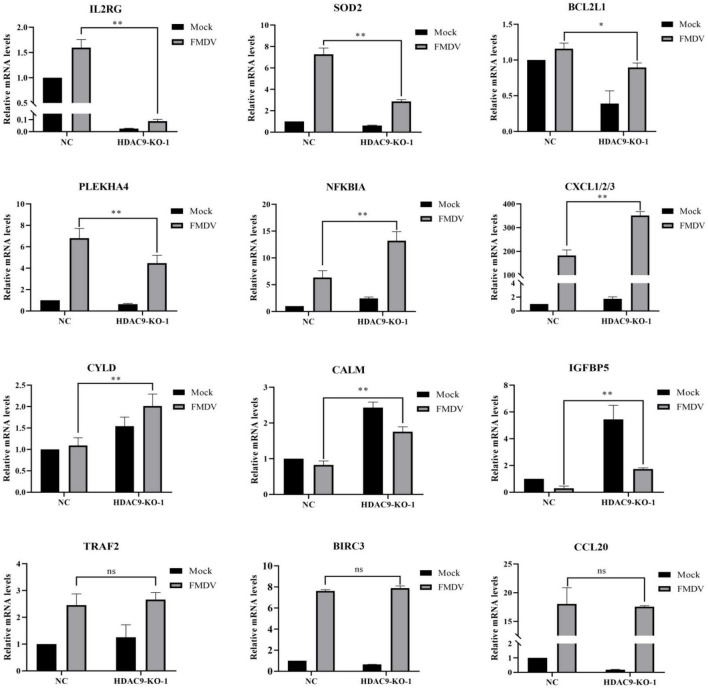
Verification of RNA-seq data with RT-qPCR. Validation of the differentially expressed genes using the RT-qPCR assay in NC and HDAC9-KO-1 cells. Experiments were performed in triplicate and repeated three times with similar results. Data are presented as mean ± SD (*n* = 3). Statistical significance was analyzed by Student’s *t*-test: *p < 0.05, **p < 0.01.

The RT-qPCR analysis was used to detect the differential gene expression to verify the accuracy of the RNA-seq data. As shown in Figure 7, the immune response-related genes were randomly selected for analysis between FMDV-infected and uninfected NC and HDA9-KO-1 cells, including IGFBP5, CALM, IL2RG, BCL2L1, PLEKHA4, CXCL1/2/3, SOD2, CCL20, BIR3, NFKIBA, TRAF2, and CYLD. The results of RT-qPCR corresponded with RNA-seq data (Figure 6C). All results indicated that HDAC9 may inhibit FMDV replication by regulating genes in these innate immunity pathways.

## Discussion

A series of cell lines, including BHK-21, PK-15, IB-RS-2 (De Castro, 1970), and SK-6 (Kasza et al., 1972) have been established as an important tool of separation, culture and research for FMDV. Propagation of FMDV by cell culture is an important method to preserve viral biological and antigenic characteristics. Currently, the production of FMD-inactivated vaccine is completely dependent on BHK-21 cells (Capozzo et al., 1997). BHK-21 cells were first established by MacPherson and Stoker using 1-day-old Syrian hamster kidney cells in 1962, which grow rapidly and have a wide sensitive spectrum of viruses, and were subsequently used for the proliferation of a variety of viruses and vaccine production (Regan and Petricciani, 1987), such as FMD disease vaccine, rabies vaccine, and Newcastle disease vaccine. However, there is still much room for improvement in viral susceptibility. The genome editing technology based on CRISPR-Cas9 has recently been applied widely to study gene function in many species. This technology has been used to modify genes that regulate the infection and immunity response of FMDV in BHK-21 cells and improve the replication efficiency of FMDV in BHK-21 cells, which will help to improve the yield or quality of FMD vaccine.

In this study, we established an HDAC9 knockout BHK-21 cell line by the CRISPR/Cas9 system. Compared with wild-type cells, these cells have a normal shape, size, and stable growth rate. Chromosome number and karyotype analysis showed that the HDAC9 knockout cell has 42 chromosomes like the wild-type BHK-21 cell and without chromosomal aberration.

HDAC9 belongs to class II HDACs, which shuttles between the nucleus and cytoplasm. In some cases, class II HDACs can also be used as transcriptional activators and results in inducible gene expression (Shakespear et al., 2011). So far, some studies have found that HDACs are the regulator of signal transduction activation in an innate immune response. HDAC9 directly maintains the deacetylation status of TBK1 and enhances its kinase activity to activate antiviral innate immunity (Li et al., 2016). We found that TRIM29 promoted DNA virus and RNA virus infections by inhibiting the innate immune response (Xing et al., 2017, 2018). TRIM29 is also called ataxia telangiectasia group D-complementing (ATDC); HDAC9 deacetylates the Lys^116^ of ATDC and changes the ability of ATDC to correlate with p53, thereby increasing the transcriptional activation function of p53 (Yuan et al., 2010). In addition, recent studies have shown that p53 inhibits innate immune response during FMD disease infection (Zhang et al., 2019). As a deacetylase, HDAC6 promotes RIG-I activation and innate antiviral immunity to recognize and restrict RNA virus infection (Liu et al., 2016). HDAC4 promotes type I interferon signaling and co-precipitate with STAT2 and restricts VACV and HSV-1 replication and spread (Lu et al., 2019). HDAC3 is an epigenetic regulator of the CD8^+^ T-cell cytotoxicity program, which inhibits CD8^+^ T cell cytotoxicity early during activation. HDAC3 was also found to modulate the signal transducer and activator of STAT3 to the LEAP-1 promoter. HDAC3 inhibitor treatment also blocked HCV replication in a mouse model of HCV infection (Zhou et al., 2018).

Our previous study found that FMDV replication was significantly higher in HDAC9 knockout cells than in NC cells. To explore the crucial role of HDAC9 in antiviral immune signaling pathways, RNA-seq was performed. We found that KEGG pathways were mainly enriched in the immune-related pathway, such as Jak-STAT, NOD-like receptor, Toll-like receptor, NF-κB, and MAPK signaling pathways. Then, multiple DEGs were selected for RT-qPCR detection. The results showed that the randomly selected genes validated by RT-qPCR displayed similar expression patterns with that in the RNA-seq data. Cytokines, including interleukin, interferons, tumor necrosis factor superfamily, colony-stimulating factor, chemokine, and growth factor, play an important role in preventing viral infection (Depalo, 2000). The transcription factor NF-κB plays a key role in coordinating the immune response. NF-κB inhibitor alpha (NFKBIA) inhibits NF-κB by blocking the nuclear localization signal of NF-κB protein and keeps it inactive in the cytoplasm (Li and Verma, 2002). We found that compared with the NC cell line, the expression of NFKBIA in the HDAC9-KO cell line was significantly upregulated by FMDV infection. During FMDV infection, the cytokines CXCL1/2/3, IL2RG, SOD2 (superoxide dismutase 2), and PLEKHA4 were upregulated by the host to stimulate inflammatory response for virus clearance. However, the expressions of these genes were reduced in the HDAC9-KO cell line compared to NC cells after FMDV infection. On the contrary, IGFPB5 (insulin-like growth factor-binding protein 5) and CALM (calmodulin) were downregulated after FMDV infection. IGFBP5 has various IGF-independent cellular activities, including receptor-independent cellular uptake followed by transcriptional regulation (Goda et al., 2008). Recently, studies have shown that the p53 gene is related to the upregulated expression of IGFPB5 during cell inflammation (Fumihiro et al., 2018). The above genes mainly regulate signal transduction, innate antiviral immune response, cell growth, and death levels, and are enriched in Toll-like receptor signaling pathway, MAPK signaling pathway, NOD-like receptor signaling pathway, NF-κB signaling pathway, and p53 signaling pathway, which indicated that HDAC9 might inhibit FMDV infection by regulating genes in these pathways.

## Conclusion

Protein acetylation and deacetylation play important roles in gene expression regulation. Acetylation or deacetylation is associated with the development of many diseases, such as cancer, neurological disorder, and inflammatory diseases. At the same time, acetylation also plays an important role in host antiviral innate immune response. In our preliminary study, HDAC9 can regulate the replication of FMDV. It is necessary to explore further the specific relationship between HDACs and FMDV, which will provide a more comprehensive understanding, theory, and application basis for the prevention and control of FMD.

## Data Availability Statement

The datasets presented in this study can be found in online repositories. The names of the repository/repositories and accession number(s) can be found below: https://www.ncbi.nlm.nih.gov/sra/PRJNA777823.

## Author Contributions

SH performed the experimental work, analyzed the data, and wrote the manuscript. SH and RM designed the experiments and assisted in manuscript preparation and revision. YS provided critical experimental design and modified the manuscript. All authors have read and agreed to the published version of the manuscript.

## Conflict of Interest

The authors declare that the research was conducted in the absence of any commercial or financial relationships that could be construed as a potential conflict of interest.

## Publisher’s Note

All claims expressed in this article are solely those of the authors and do not necessarily represent those of their affiliated organizations, or those of the publisher, the editors and the reviewers. Any product that may be evaluated in this article, or claim that may be made by its manufacturer, is not guaranteed or endorsed by the publisher.

## Footnotes

1
http://crispor.tefor.net/

2
https://www.ncbi.nlm.nih.gov/sra/PRJNA777823
